# An Efficient TetR/TetO-Integrated Packaging System for Fowl Adenovirus 4 Vector Carrying Toxic Transgene

**DOI:** 10.3390/mps9030100

**Published:** 2026-06-22

**Authors:** Qian-Wen Ma, Zhi Li, Zhi-Chao Zhang, Xiao-Juan Guo, Xiao-Hui Zou, Tao Hung, Zhuo-Zhuang Lu

**Affiliations:** 1 NHC Key Laboratory of Medical Virology and Viral Diseases, National Institute for Viral Disease Control and Prevention, Chinese Center for Disease Control and Prevention, Beijing 100052, China; maqw2021@163.com (Q.-W.M.); 14747811207@163.com (Z.L.); zzz-em@sina.com (Z.-C.Z.); guoxj@ivdc.chinacdc.cn (X.-J.G.); zouxh@ivdc.chinacdc.cn (X.-H.Z.); hongt@cae.cn (T.H.); 2 School of Public Health, Baotou Medical College, Inner Mongolia University of Science and Technology, Baotou 014040, China

**Keywords:** LMH cell, colony forming, fowl adenovirus 4, packaging, tetracycline repressor

## Abstract

Adenoviral vectors are widely used for gene therapy and vaccine development. To circumvent pre-existing immunity against commonly used human adenovirus type 5, vectors based on rare human serotype or animal adenoviruses have attracted increasing interest. Previously, we constructed vectors based on fowl adenovirus 4 (FAdV-4) and replaced the knob of FAdV-4 fiber2 with that of FAdV-1 fiber1 to generate FAdV4-CF1K vectors with enhanced transduction efficiency in human cells. In this study, we aimed to modify the packaging system to efficiently produce FAdV-4 vectors carrying transgenes toxic to viral replication. Chicken LMH cells failed to form colonies at low seeding densities. We collected used medium from LMH cell cultures and used it as a supplement to adapt LMH cells, generating the colony-competent subclone LMH-C3532. A lentiviral vector encoding a codon-optimized tetracycline repressor (tetR) was transduced into LMH-C3532 to establish a tetR-integrated cell line, LMH-tetR24. An adenoviral plasmid, pKFAV4-CF1K-CtG, was constructed in which a tetracycline operator (tetO)-bearing CMV promoter controlled GFP expression. The SwaI-flanked GFP in this plasmid was replaced with the HA gene from an H5N1 influenza virus to generate pKFAV4-CF1K-CtHA. Linearized adenoviral plasmids were transfected into LMH-tetR24 cells, and recombinant FAdV4-CF1K-CtG and FAdV4-CF1K-CtHA viruses were successfully rescued, amplified, and purified. When infected with FAdV4-CF1K-CtG at various multiplicities of infection (MOI), the progeny virus yield from LMH-tetR24 cells was 4–10 times higher than that from LMH-C3532 cells. For FAdV4-CF1K-CtHA, the yield difference between the two cell lines was even more pronounced, reaching 3–4 orders of magnitude. Overexpression of HA in LMH-C3532 cells negatively affected FAdV4-CF1K-CtHA replication, resulting in smaller and fewer plaques. In conclusion, by separately integrating tetR into packaging cells and TetO into the adenoviral plasmid, we established a system that can be routinely used to package FAdV-4 vectors. Notably, this system facilitates the propagation of FAdV-4 vectors carrying toxic transgenes.

## 1. Introduction

Adenoviral vectors are widely used in basic biological research [1], gene therapy [2], and vaccine development [3,4,5]. Human adenovirus type 5 (HAdV-5) has been studied in-depth as a protovirus, and most adenoviral vectors are constructed based on HAdV-5 genome. The ecosystem for construction of HAdV-5 vectors has fully developed [6,7], many techniques have been established, and related materials are even commercially available. However, high prevalence of serum antibody against HAdV-5 hampers the application of HAdV-5 vectors in human gene therapy and vector vaccine because pre-existing immunity restricts vector transduction and leads to reduced transgene expression. Recently, interest has been attracted to vector systems based on animal adenoviruses or human adenoviruses of rare serum type [6,8].

A vector system based on fowl adenovirus 4 (FAdV-4) was established in the laboratory previously, and a chicken cell line named LMH (Leghorn Male Hepatoma) was used as the packaging cell [9]. We attempt to use it as a gene transfer tool considering that humans lack the pre-existing immunity against FAdV-4 since FAdV-4 cannot infect human beings. To improve the gene transfer efficiency of FAdV-4 vector to human cells, the knob domain of FAdV-4 fiber2 was replaced with that of FAdV-1 fiber1 to generate a fiber-chimeric FAdV-4 vector, which we named FAdV4-CF1K [10]. To validate if such vector could be used as vaccine vector for mammals, an FAdV-4 adenoviral plasmid was constructed, in which HA gene of H5N1 influenza virus was inserted downstream of CMV promoter. Recombinant virus was rescued successfully. However, the virus yield was very low, and the virus band was barely visible after purification by density ingredient ultracentrifugation. We assumed that it might result from the negative effect of overexpressed HA protein on the replication of FAdV-4 in chick cells, and minimization of HA expression in packaging cells would help improve virus yield.

We attempted to integrate the tetracycline (tet)-regulated gene expression strategy into the FAdV-4 vector construction system to reduce the transgene expression in packaging cells during recombinant virus amplification. Tet-controlled gene expression systems are constructed based on regulatory elements that control the activity of the tet-resistance operon in bacteria, where tet repressor (tetR) proteins form dimers that recognize and bind the tet operator (tetO) cis-regulatory element, thus suppressing the activity of the promoters and shutting off related protein production in the absence of tet [11]. While in the presence of tet, binding of tet triggers a conformational change in the tetR dimer that prevents tetO binding, which will release the activity of tetO-bearing promoter and lead to transcription of the downstream gene. Yao F et al. inserted two tandem tetO sequences 10 bp downstream of the TATA box of CMV promoter and used this tetO-fused CMV promoter to control the expression of transgene in mammal cells [12]. In the presence of tetR protein and with no tet, the activity of CMV promoter was suppressed and the expression of transgene was inhibited. When there was no tetR, the tetO-fused CMV promoter worked just like original CMV promoter and the transgene was highly transcribed. This strategy would be employed in this study.

During the selection of genetically modified LMH cell lines, we observed that LMH cells failed to form colonies when seeded at a low density. Therefore, in this study, we first adapt LMH cell line to enhance its colony-forming ability, and then attempted to establish a subclone of LMH to constitutively expressing tetR protein, followed by constructing a FAdV-4 vector carrying tetO-fused CMV promoter to control the expression of a transgene.

## 2. Materials and Methods

### 2.1. Plasmids, Primers, and Molecular Cloning

Plasmids of pCDH-CMV-MCS-EF1-copGFP (System Biosciences, Palo Alto, CA, USA; Cat# CD511B-1), psPAX2 and pMD2.G (a gift from Didier Trono; Addgene, Watertown, MA, USA; plasmids #12259 and #12260) were used for lentivirus vector construction and packaging. Plasmids of pcDNA4toMyc-HisA (Thermo Fisher Scientific, Waltham, MA, USA) and pLEGFP-C1 were used to clone tetO-fused CMV promoter and GFP gene, respectively. pKFAV4S-F2CF1K-CG is a previously constructed FAdV-4 adenovirus plasmid [10]. It carries deletions of ORF0, ORF1, ORF1B, ORF2 and ORF19A in FAdV-4 genome; the knob coding sequence (CDS) of fiber2 was replaced with that of fiber1 from FAdV-1 (CELO virus) to enhance the gene transduction to mammalian cells; and at the left deletion site in the genome, a CMVp controlled GFP expression cassette was inserted, which was flanked with two SwaI sites to facilitate transgene replacement.

DNA oligonucleotides were designed, synthesized and used as PCR primers (Table 1). PCR or overlap extension PCR were routinely performed for gene cloning (Q5 High-Fidelity DNA Polymerase; New England Biolabs, Ipswich, MA, USA; Cat. M0491S) or plasmid identification (Premix Taq; TaKaRa, Dalian, China; Cat. RR901A). Plasmid construction was performed with methods of Gibson assembly (NEBuilder HiFi DNA Assembly Master Mix; New England Biolabs, Ipswich, MA, USA; Cat. E2621) or restriction-ligation cloning (DNA Ligation Kit; TaKaRa, Kusatsu, Japan; Cat. 6022Q). Restriction enzymes were purchased from New England Biolabs or TaKaRa. DNA recovery and cleaning were performed with kits from Zymo Research (Zymo Research, Irvine, CA, USA; Cat. D4045 and D4010). Escherichia coli TOP10 chemically competent cells were used for plasmid transformation according to the heat shock procedure described in the manufacturer’s instructions (TIANGEN Biotech, Beijing, China, Cat. CB104-02).

### 2.2. Cell Culture

Chicken hepatoma cell line LMH (American Type Culture Collection, ATCC CRL-2117), its derivatives (LMH-C3532 and LMH-tetR24), 293 cells (ATCC CRL-1573) and 293T cells (ATCC CRL-3216) were maintained in Dulbecco’s modified Eagle’s medium (DMEM; HyClone, Logan, UT, USA; Cat. SH30022.01) plus 10% fetal bovine serum (FBS; HyClone, Logan, UT, USA; Cat. SV30208.02) at 37 °C in a humidified atmosphere supplemented with 5% CO_2_, and routinely passaged twice a week. Before adaptation, original LMH was cultivated on flasks or plates pretreated with 0.1% gelatin according to the instruction of ATCC. For virus rescue or amplification, exponentially growing cells were split at a ratio of 1:3, transfection or infection were carried out one day later. After incubation for indicated time, the old culture medium was discarded and replaced with fresh DMEM containing 2% FBS.

### 2.3. Construction of Lentivirus Vector

Self-inactivated lentivirus vector was constructed to express tetR protein. The CDS of tetR protein (GenBank WP_000088605) was generated by codon-optimizing tool iCodon [13] and synthesized (Supplementary Document). EF1a promoter fragment was PCR amplified (Table 1). Plasmid pCDH-CMV-MCS-EF1-copGFP was digested with restriction enzymes of ClaI and SalI, and transgene-removed fragment of 5828 bp in length was recovered from agarose gel after electrophoresis. EF1a promoter, tetR CDS and the backbone of lentivirus vector were subjected into Gibson assembly to generate plasmid pCDH-EF1a-tetR. Another lentivirus plasmid of pCDH-CMVtGFP was similarly constructed, in which the expression of GFP was controlled by a tetO-bearing CMV (CMVt) promoter. The CMVt promoter and GFP CDS were amplified by PCR (Table 1), which were inserted into the ClaI/SalI site of pCDH-CMV-MCS-EF1-copGFP to generate pCDH-CMVtGFP by Gibson assembly.

Lentiviral vectors of CDH-EF1a-tetR or CDH-CMVtGFP were produced in 293T cells after transfection of pCDH-EF1a-tetR or pCDH-CMVtGFP with the helper plasmids of psPAX2 and pMD2.G. The vector-containing culture media were collected, centrifugated to remove cellular debris, preserved at −80 °C and titrated on 293 cells by determining the copy number of integrated provirus in cellular genome with real-time PCR after virus infection (Table 1) [14,15].

### 2.4. Reverse Transcription Quantitative PCR

Reverse Transcription Quantitative PCR (RT-qPCR) was performed to confirm the transcription of tetR in LMH-tetR24 cells. Briefly, exponentially growing cells in 6-well plates were lysed with TRIzol reagent (Thermo Fisher Scientific, Waltham, MA, USA; Cat. 15596018), total RNA was extracted, quantified and reverse-transcribed to cDNA (SuperScript IV VILO Master Mix, Thermo Fisher Scientific, Waltham, MA, USA; Cat. 11766050; genomic DNA removal was integrated in this step). SYBR qPCR was performed to amplify fragments of tetR or chicken β-actin genes with primer pairs of 2606tetRFf/r or 1905C-ActBf/r by using cDNA as the template (SupRealQ Ultra Hunter SYBR qPCR Master Mix U+, Vazyme Biotech, Nanjing, China; Cat. Q713). Melt curve plots were inspected to ensure specific amplification at the end of the PCR procedure, and the PCR products were further resolved by agarose gel electrophoresis.

### 2.5. Construction of FAdV-4 Adenoviral Vectors

The expression cassette of CMV promoter controlled GFP in pKFAV4S-F2CF1K-CG was replaced with that of CMVt promoter controlled GFP to generate pKFAV4-CF1K-CtG plasmid. CMVt promoter and GFP fragments were amplified by PCR and fused to one fragment by overlap-extension PCR (Table 1), followed by being inserted to the SwaI sites in pKFAV4S-F2CF1K-CG to generate pKFAV4-CF1K-CtG by Gibson assembly. In pKFAV4-CF1K-CtG, the GFP CDS was flanked with SwaI dual cutter sites, which would facilitate the replacement of transgene. HA gene of a H5N1 influenza virus was synthesized (GenBank PP577943), amplified by PCR (Table 1) and used to replace GFP in pKFAV4-CF1K-CtG to generate pKFAV4-CF1K-CtHA plasmid by Gibson assembly.

### 2.6. Rescue, Purification and Titration of Adenoviral Vectors

FAdV-4 adenovirus plasmid was linearized by PmeI digestion, recovered, mixed with jetPRIME reagents (Polyplus-transfection, Illkirch, France; Cat. 114-15) and used to transfect LMH-tetR24 cells. Recombinant adenovirus was rescued, amplified in LMH-tetR24 cells, and purified by Iodixanol density gradient ultracentrifugation. The virus particle titer (vp/mL) was determined by measuring the content of virus genomic DNA using the Qubit double-stranded DNA assay kit (Thermo Fisher Scientific, Waltham, MA, USA; Cat. Q32851). The multiplicity of infection (MOI) was calculated from particle titers. The details of these experiments could be found elsewhere [10]. Infectious titer was determined on LMH cells by limiting dilution assay. In brief, LMH cells were seeded in 96-well plates, and 10-fold dilutions of virus were added to the adherent LMH cells one day later. For FAdV4-CF1K-CtG (CF1K-CtG for short) virus, GFP-positive cells were counted under fluorescence microscope (Leica DMi8 microscope; Leica Microsystems, Wetzlar, Germany) at 36 h post infection, and the infectious titer (IU/mL) was calculated as average number of GFP+ cells per well × dilution factor/volume of virus suspension per well. For FAdV4-CF1K-CtHA (CF1K-CtHA for short) virus, immunofluorescence assay for HA expression (see Section 2.8) was performed at 36 h post infection, HA-positive cells were counted and the infectious titer (IU/mL) was similarly calculated; or the number of wells where plaques occurred at the highest dilution were counted under microscope 6 days post infection, and the infectious titer (PFU/mL) was calculated as number of plaque-positive wells × dilution factor/(volume of virus suspension per well × number of wells for each dilution). It turns out that the titer values of CF1K-CtHA determined by these two methods were very close, and we routinely used the PFU method to titrate unpurified CF1K-CtHA.

### 2.7. Preparation and Restriction Analysis of Adenovirus Genomic DNA

Purified recombinant FAdV-4 was mixed with an equal volume of 2 × lysis solution (20 mM EDTA, 0.2% SDS, 0.4 mg/mL proteinase K, pH 7.4) and incubated at 50 °C for 2 h. The genomic DNA was recovered by using a DNA cleaning kit, digested with selected restriction enzymes, resolved on 0.7% agarose gel by electrophoresis and photographed. Original adenoviral plasmid was similarly digested and used as a control.

### 2.8. Immunofluorescence Assay

Exponentially growing 293 cells were seeded on a 35-mm dish. After incubation for 1 day, the cells were infected with CF1K-CtHA at an MOI of 1000 vp/cell for 2 h. At 48 h post infection, the culture medium was aspirated and the cells were fixed in 4% paraformaldehyde in PBS for 15 min at room temperature. After rinse with PBS-T buffer (PBS containing 0.1% Tween-20), the cells were permeabilized with 0.3% Triton X-100 in PBS containing 1% bovine serum albumin (BSA) for 60 min, followed by incubation with 1:200 diluted anti-HA (Influenza A H5N1) primary antibody (SinoBiological, Beijing, China; Cat. 11048-RM09) for 2 h at room temperature. The cells were rinsed with PBS-T for 3 times, incubated with the 1:200 diluted FITC-labelled secondary antibody and 1:2000 diluted DAPI (Thermo Fisher Scientific, Waltham, MA, USA; Cat. D1306) in PBS-T for 1 h, rinsed with PBS-T for 3 times, mounted with a drop of mounting medium (Solarbio, Beijing, China; Cat. S2100) and photographed under a confocal microscope. Uninfected 293 cells served as a negative control.

### 2.9. Adaptation of LMH Cells for Enhanced Colony-Forming Ability

Exponentially-growing LMH cells were detached from the flask with 0.15% trypsin in phosphate-buffered saline (PBS), counted, diluted in indicated culture medium, seeded to wells of 6-well plate with or without gelatin pretreating in numbers of 800, 400, 200, 100, 50 and 25 cells per well, and cultured in CO_2_ incubator for 2 weeks. Wells where separated cell colonies grew were selected for colony picking by using the cloning ring technique [16]. The cloned cells were amplified, and these that tended to grow to monolayer without aggregation were subjected to the next round of colony selection.

### 2.10. Selection of LMH Cells Expressing Functional tetR

Subcloned LMH cells with increased colony-forming ability (LMH-C3532) were 1:3 split and seeded on wells of 24-well plate. At 16–24 h post seeding, when cells reached 70% confluency, lentivirus aliquot of CDH-EF1a-tetR in 0.2 mL was mixed with 0.2 mL fresh culture medium, and polybrene was added to a final concentration of 8 µg/mL. After removal of the old culture medium, the diluted virus was added to the cells to reach an MOI of 20 IU/cell. The plate was centrifugated at 1000× *g* for 1 h at room temperature before being transferred to the incubator. The virus-containing medium was aspirated and fresh medium was supplemented at 12 h post infection. At 48 h post infection, the cells were detached, counted, diluted and seeded to 6-well plate for growing cell colonies as described in 2.9. For the first round, 15 cell colonies were picked, amplified and used for functional screening.

Amplified cells from each colony were passaged to 2 wells of 48-well plates, lentivirus of CDH-CMVtGFP was added at a dose of 20 µL/well to reach an MOI of 4 IU/cell), and polybrene was added to a final concentration of 8 µg/mL. GFP+ cells were photographed by using a cell culture scanner (Countstar Castor, Countstar, Shanghai, China) at 24 h post infection. After GFP fluorescence scanning, old medium was removed. For the 2 replicate wells, 0.2 mL fresh medium was added to one well, while 0.2 mL fresh medium containing 1 µg/mL doxycycline (Dox) was added to the other. GFP fluorescence were photographed and analyzed by using the cell culture scanner for the second time at 48 h post infection. Fold induction was calculated as the ratio of GFP+ cell percentage times mean fluorescence intensity in well with Dox to that in well without Dox. Increased expression of GFP after the addition of Dox indicated that the cloned cells had functional tetR (fold induction was greater than 1), and such cloned cells were subjected to another round of subclone screening.

### 2.11. Evaluate Virus Packaging Ability of LMH-tetR24 Cells

LMH-C3532 or LMH-tetR24 cells in 100 µL culture medium were seeded in 96-well plates and used for virus infection when they reached 70% confluency the next day. CF1K-CtG or CF1K-CtHA were ten-fold serially diluted in DMEM plus 2% FBS, and viruses with a dose of 4 × 10^1^ to 4 × 10^5^ vp in a volume of 10 µL was added to each well. Eight replicates were set. At 36 h post infection, GFP expression and/or cell confluency were analyzed by using the cell culture scanner. Six days post infection, the plates were subjected to 3 rounds of freeze-and-thaw, lysates from 4 of the 8 replicate wells were combined and spin to remove cellular debris. Progenitor viruses in the supernatant were titrated on LMH-C3532 cells.

### 2.12. Plaque Forming Experiment

LMH-C3532 or LMH-tetR24 cells in 2 mL culture medium were seeded in 6-well plates. One day later, purified CF1K-CtG or CF1K-CtHA were diluted and added to the wells at a dose of 3000 vp/well. After 5 h of absorption, the old medium was aspirated, the cells were washed with DMEM, and 2.5 mL semisolid culture media containing 2% FBS and 1% low-melting agarose were added to each well. After incubation for 6 days, 2 mL 4% paraformaldehyde in PBS was added to each well, and the cells were fixed for 2 h at room temperature. The semisolid culture media were removed, and the adherent cells on the bottom were stained with crystal violet solution. The plates were photographed, size of the plaques was measured by using the Fiji image processing package (http://fiji.sc/ accessed on 1 March 2021), and the sizes of the plaques formed on different cell lines were compared by using the Mann-Whitney nonparametric test.

### 2.13. Lentivirus Integration Sites Analysis

The genomic DNA was extracted after LMH-tetR24 cells have been passaged twenty times (TIANamp Genomic DNA Kit; TIANGEN BIOTECH, Beijing, China; Cat. DP304), the integration of CDH-EF1a-tetR provirus was confirmed by real-time PCR (Table 1), and the whole genome sequencing (WGS) was performed to find the integration sites of lentivirus CDH-EF1a-tetR. The clean 150 bp paired-end data with a sequencing depth of 50× were released to the laboratory, and the pipeline of nf-core/viralintegration was set up to analyze the lentivirus integration (https://github.com/nf-core/viralintegration, accessed on 1 March 2026) [17].

### 2.14. Statistical Analysis

Data were presented as the mean ± standard deviation (SD) of triplicate measurements from a representative experiment, which was repeated at least twice with similar results, unless otherwise indicated. The statistical analysis was performed using the regular one-way or two-way analysis of variance (ANOVA) test. Following one-way ANOVA, post hoc multiple comparisons were performed using Tukey’s HSD test to determine differences between all pairs of groups. Sidak’s multiple comparisons test was performed for the main effects after a two-way ANOVA was conducted. The data of fluorescence intensity and virus yield were log-transformed before the statistical analysis. A *p*-value less than 0.05 was considered to be significant.

## 3. Results

### 3.1. LMH Cells Failed to Form Colonies in Regular Cell Culture Medium

**Figure 1:**
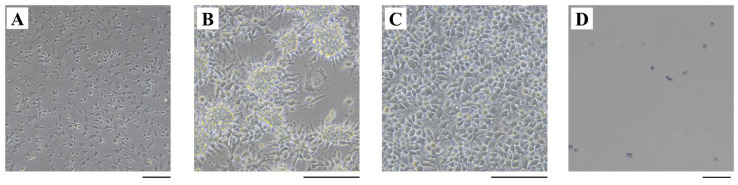
Morphology of LMH cells cultured under different conditions. (**A**). Twenty-four hours post-seeding, LMH cells cultured on regular flasks (without gelatin pretreatment) displayed a fully extended polygonal morphology. (**B**). Three days after seeding, LMH cells cultured on regular flasks aggregated and formed local multilayers. (**C**). Three days after seeding, LMH cells cultured on gelatin-pretreated flasks grew uniformly and formed a monolayer. (**D**). When LMH cells were seeded at low density on gelatin-pretreated flasks, they initially attached and displayed polygonal morphology. However, they failed to proliferate. Instead, they gradually rounded up, detached, and ultimately degenerated. Shown are rounded cells 7 days after seeding. Scale bar: 100 μm.

LMH cells were dispersed into single cells after trypsin treatment, and scattered cells adhered to the inner surface of the flask in DMEM plus 10% FBS in 2 h (Figure 1A). As the culture time elongated, the cells aggregated and clumped up although there still was adequate extra space for them to grow on (Figure 1B). If the flask was pretreated with gelatin as ATCC suggested, the cells would grow and form a monolayer (Figure 1C). However, if the cells were seeded at a low density, for example, 1 × 10^4^ LMH cells were plated on a gelatin-pretreated T25 flask, the cells stopped dividing and died gradually (Figure 1D). Single LMH cells are unable to grow into colonies in regular culture condition, which hampers the establishment of LMH cell lines to stably express transgene.

### 3.2. The Used Culture Medium Promoted the Growth of LMH Cells

**Figure 2:**
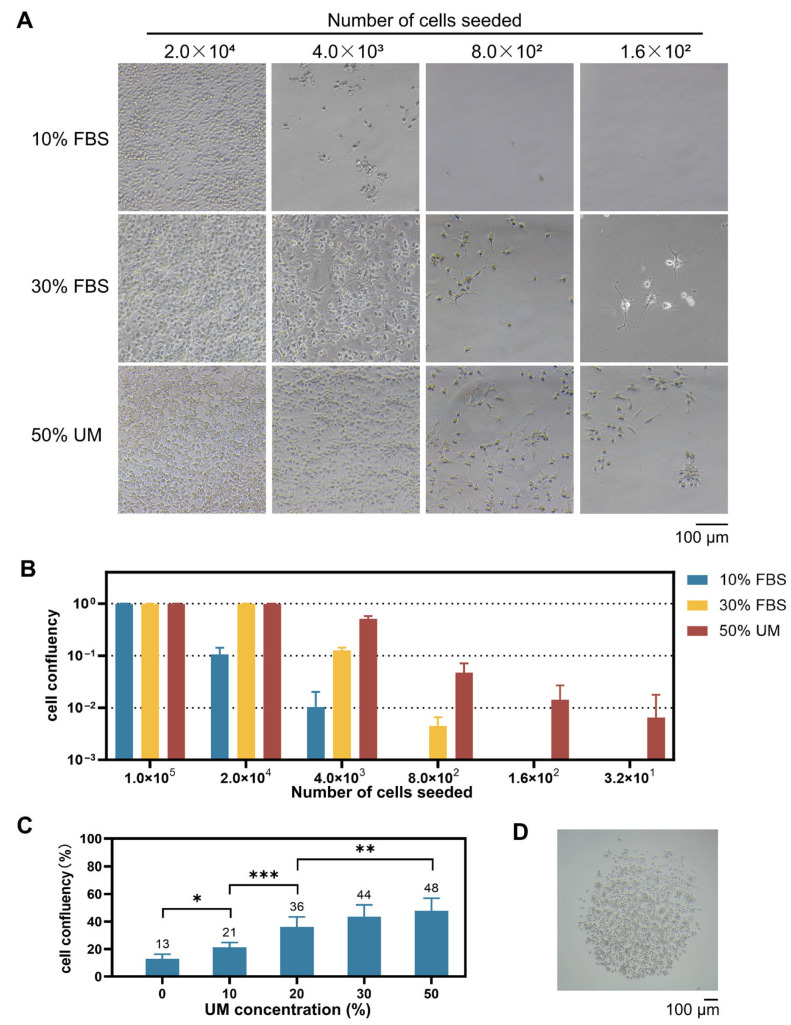
Effect of the used medium on the growth of LMH cells. Exponentially growing LMH cells were split as a ratio of 1:3 and cultured for 2 days. The used medium (UM) was then harvested and used as a supplement for subsequent LMH culture. Five serial dilutions of LMH cells were cultured in DMEM containing 10% FBS, 30% FBS, or 10% FBS plus 50% UM. The cells were observed under microscope and photographed (**A**), and the cell confluency was analyzed by using a cell scanner (**B**). A dose-response relationship was observed between the UM and LMH cell growth (**C**). LMH cells were seed at a low density and culture in DMEM containing 10% FBS plus 30% UM, and cell colonies were observed after incubation for 15 days (**D**). * *p* < 0.05, ** *p* < 0.01, and *** *p* < 0.001.

Used culture medium and FBS were evaluated for their effects on cell growth. LMH cells were routinely split at a ratio of 1:3 and cultivated on gelatin-pretreated flasks in the medium of DMEM containing 10% FBS. Two days later, the culture medium was harvested, passed through 0.45 µm filter, stored at 4 °C or −80 °C and used as a supplementary ingredient for LMH cultivation, which we called used-medium (UM). Cell suspension was five-fold serially diluted, seeded in 24-well gelatin-pretreated plates and cultivated in three types of DMEM medium: plus 10% FBS, plus 30% FBS or plus 10% FBS/50% UM. After incubation for 6 days, the cells were photographed (Figure 2A), and the cell confluency was analyzed (Figure 2B). It could be seen that the cells kept growing when the number of seeded cells decreased to 32 or even 5 if 50% UM was supplemented. Increase in FBS concentration promoted the proliferation of LMH cells. However, when the number of seeded cells reduced to 32, no LMH cells could survive even if 30% FBS was added. The concentration of UM was further optimized. As shown in (Figure 2C), 10% UM could promote cell growth significantly, and 20% or 30% should be the appropriate concentration considering that DMEM nutrients in UM had been partially consumed.

### 3.3. Adaptation of LMH Cells to Enhance the Colony-Forming Ability

We aimed to adapt LMH to form colonies in regular culture medium on flasks without gelatin treatment. First, LMH cells were split at a radio of 1:100, seeded in flask without gelatin treatment and cultivated in DMEM plus 10% FBS/30% UM. After incubation for 8 days, the cells reached a confluency of 80%, and the passaging experiment was repeated once to make the cells adapt to grow at a relatively low density. After that, 1000 cells were seeded in 6-well plate and cultivated in medium containing 30% UM for 15 days. Several colonies could be found (Figure 2D), and they were picked up, transferred to 24-well plates and given identifier LMH-C1 (clone #1), LMH-C2 and so on. LMH-C3 grew into a monolayer with the best cellular morphology, and it was subcloned and LMH-C35 was selected and proliferated. LMH-C35 cells (1 × 10^4^) were seeded in T25 flask and cultivated in DMEM plus 10% FBS without the addition of UM. These cells attached to the bottom sparsely at the beginning, and then they entered a crisis phase characterized by extensive cell death in the absence of UM. The dead cells were removed by changing culture medium a week post seeding. After incubation for 4 weeks, some cells survived and formed colonies. These cells were detached, combined and subjected to another two rounds of subcloning. Finally, a subclone of LMH, LMH-C3532, was obtained. In contrast to the parent LMH, LMH-C3532 could grow to monolayer on ordinary flasks in regular medium of DMEM plus 10% FBS. If 100 cells of LMH-C3532 were seeded to a well of 6-well plate, approximately 10 colonies could form after incubation for 2 weeks. On the other hand, the yield of progeny virus in LMH-C3532 was as much as that in LMH if the same dose of FAdV-4 seed virus was used.

### 3.4. Establishment of tetR-Integrated LMH Cell Lines

**Figure 3:**
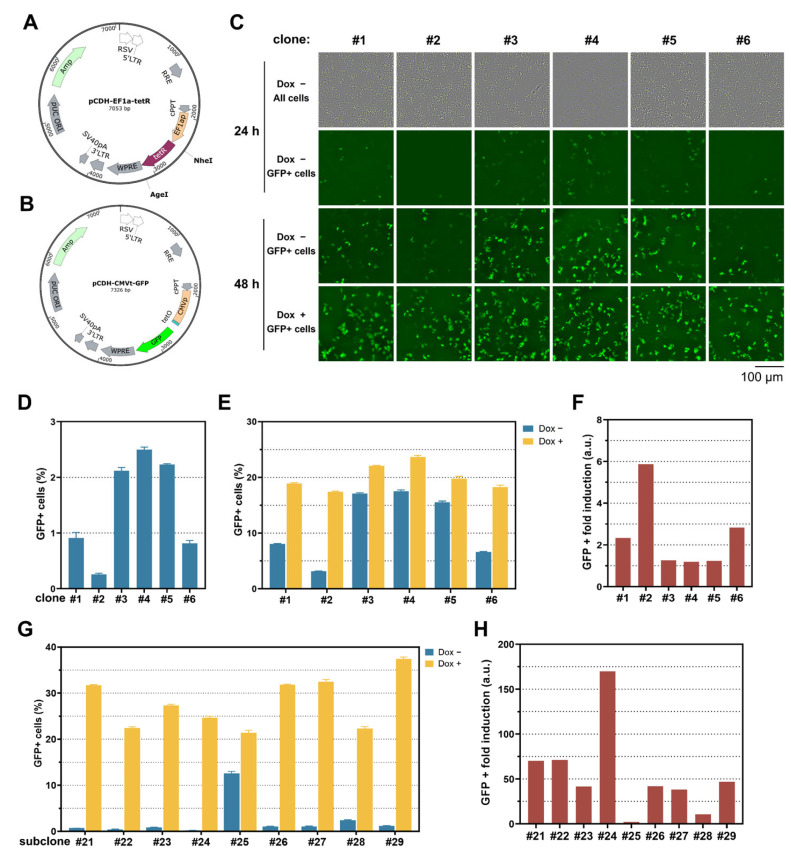
Selection of LMH cell lines that expressed functional tetR. LMH-C3532 cells were infected with lentivirus vector carrying EF1a promoter-controlled tetR gene (CDH-EF1a-tetR). The infected cells (LMH-tetR) were detached, serially diluted, seeded in 6-well plate and used to grow colonies. After incubation for 2 weeks, cell colonies were picked, proliferated, and subjected to functional screening. The cloned cells (LMH-tetR1–LMH-tetR6) were passaged to wells in duplicate and infected lentivirus vector carrying tetO-bearing CMV (CMVt) promoter-controlled GFP (CDH-CMVt-GFP). Plasmid map of the lentiviral vector carrying EF1a promoter controlled tetR gene (**A**). Plasmid map of the lentiviral vector in which the expression of GFP gene is controlled under tetO-bearing CMV (CMVt) promoter (**B**). Dox was added to one of the duplicate wells at 24 h post infection. GFP+ cells were observed under a fluorescence microscope at 24 and 48 h post infection (**C**). The expression of GFP was quantitively assayed by using a cell scanner at 24 (**D**) and 48 (**E**) hours post infection. The fold induction was defined as the ratio of (percentage of GFP+ cells × mean fluorescence intensity of GFP+ cells) in Dox-treated wells to that in corresponding untreated wells (**F**). Clone LMH-tetR2 was selected and subcloned to eliminate cross-colony cell contamination. The expression of GFP (**G**) and the fold induction (**H**) was then similarly assayed after Dox addition.

Lentiviral plasmids were constructed based on the backbone of a self-inactivating vector (Figure 3A,B), and lentiviruses were prepared by the method of three-plasmid co-transfection to 293T cells. Lentivirus CDH-EF1a-tetR contained a single expression cassette of human EF1a promoter-controlled codon-optimized tetR gene, while the expression of GFP was controlled under tetO-bearing CMV (CMVt) promoter in CDH-CMVt-GFP.

**Figure 4:**
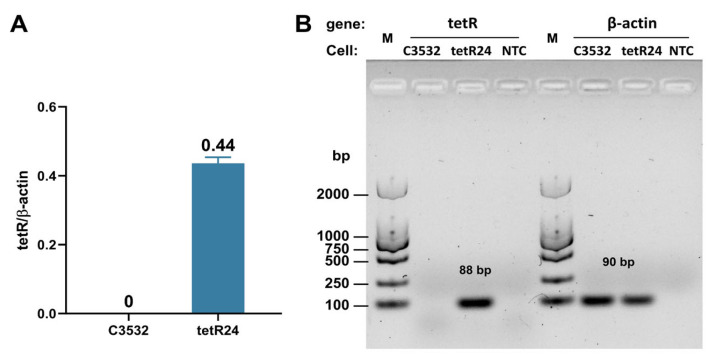
Detection of tetR expression in LMH-tetR24 cells by reverse transcription Quantitative PCR (RT-qPCR). RNA in LMH-tetR24 cells (passage 30) was extracted, reverse-transcribed and used as the template for SYBR qPCR to detect the transcription of tetR24 gene as well as internal control β-actin gene. RNA extracted from LMH-C3532 cells served as a control. The transcription of tetR gene was presented as the ratio of the copy number of tetR to that of β-actin (**A**). The PCR products were further resolved on agarose gel by electrophoresis (**B**). C3532: LMH-C3532; tetR24: LMH-tetR24; NTC: no template control; and M: DL2000 DNA marker.

LMH-C3532 cells were infected with CDH-EF1a-tetR or CDH-CMVt-GFP. Two days post infection, cells were detached, dispersed into single cells, serially diluted, seeded in 6-well plate and cultivated for 2 weeks. Approximately 90% colonies expressed GFP when being observed under fluorescence microscope for these formed by CDH-CMVt-GFP-infected LMH-C3532 cells. Colonies formed by CDH-EF1a-tetR-infected cells were picked, amplified, and subjected into functional selection after being infected with CDH-CMVt-GFP. For the first batch of 6 colonies, colony #2 (LMH-tetR2) had the lowest percentage of GFP+ cells 24 h post infection, followed by #6 and #1 (Figure 3C,D). After addition of the inducer Dox, the percentage of GFP+ cells increased for all colonies, with the greatest increase of GFP expression being observed in LMH-tetR2 (Figure 3E,F). In other 2 batches, LMH-tetR7 and LMH-tetR11 were selected, respectively. LMH-tetR2 was chosen for subcloning, followed by functional assay of tetR. GFP expression was enhanced after Dox addition for all subcloned cells, while LMH-tetR24 had the highest fold induction (Figure 3G,H). LMH-tetR24 cells were amplified, preserved and used in the following experiments. The transcription of tetR gene in LMH-tetR24 was confirmed by RT-qPCR (Figure 4A,B).

### 3.5. Preparation of FAdV-4 Vectors Carrying CMVt Promoter-Controlled Expression Cassette

**Figure 5:**
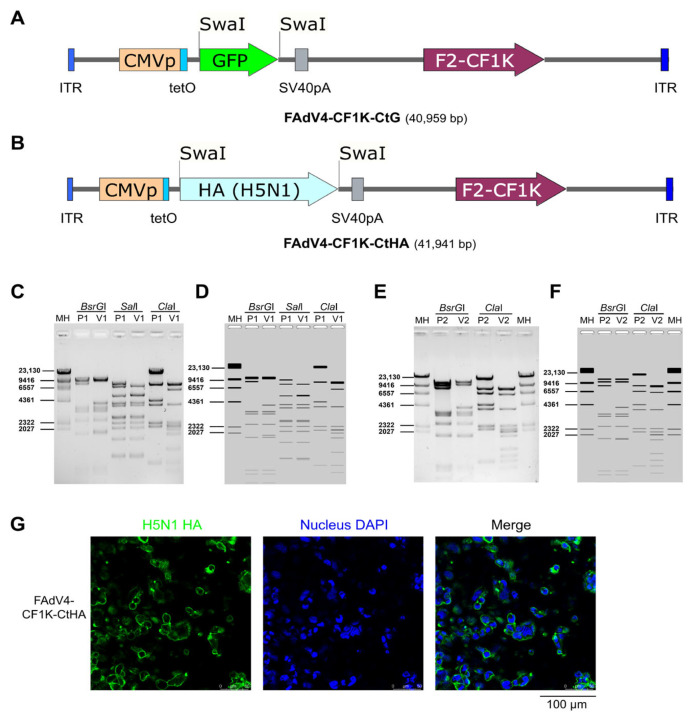
Construction and identification of FAdV-4 vectors. (**A**). Schematic diagram of FAdV4-CF1K-CtG genome, in which the expression cassette of tetO-bearing CMV promoter (CMVt) controlled GFP gene was inserted and the coding sequence of fiber 2 knob was replaced with that of CELO fiber1 knob (F2-CF1K). (**B**). Schematic diagram of FAdV4-CF1K-CtHA genome, which was generated by replacing the coding sequence of GFP in FAdV4-CF1K-CtG with that of HA from a H5N1 influenza virus. (**C**). Restriction analysis of FAdV4-CF1K-CtG genome (V1), and adenoviral plasmid pKFAV4-CF1K-CtG (P1) was used as a control. (**D**). Stimulated DNA electrophoresis for digested FAdV4-CF1K-CtG genome (V1) and pKFAV4-CF1K-CtG (P1) by using software. (**E**). Restriction analysis of FAdV4-CF1K-CtHA genome (V2), and adenoviral plasmid pKFAV4-CF1K-CtHA (P2) was used as a control. (**F**). Stimulated DNA electrophoresis for digested FAdV4-CF1K-CtHA genome (V2) and pKFAV4-CF1K-CtHA (P2) by using software. (**G**). Detect the expression of HA on FAdV4-CF1K-CtHA-infected 293 cells 2 days post infection by immunofluorescence assay.

TetO-bearing CMV promoter was used to replace original CMV promoter to generate adenoviral plasmid, and recombinant adenoviruses, CF1K-CtG and CF1K-CtHA were rescued and amplified in LMH-tetR24 cells (Figure 5A,B). The information of purified viruses was summarized in Table 2. The genomic DNA of purified viruses were identified by restriction analysis (Figure 5C–F). The expression of HA gene was confirmed in CF1K-CtHA-infected 293 cells by immuno-fluorescence assay (Figure 5G).

### 3.6. Evaluate the Replication of CF1K-CtG in LMH-tetR24 Cells

**Figure 6:**
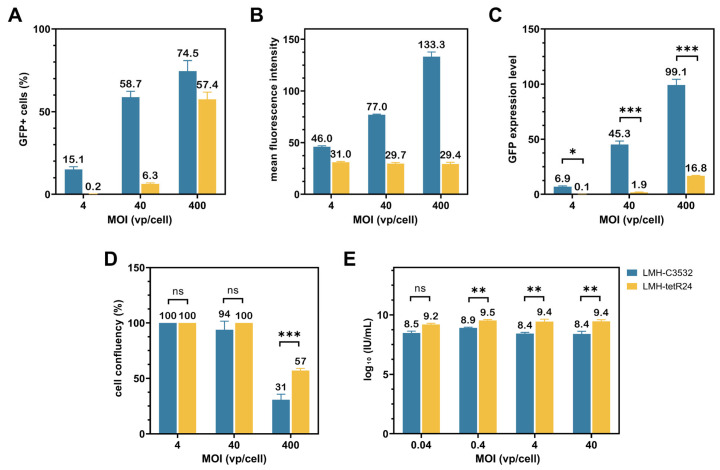
Amplification of FAdV4-CF1K-CtG in LMH-C3532 and LMH-tetR24 cells. Cells were infected with purified FAdV4-CF1K-CtG at serial MOIs. At 36 h post infection, the GFP expression and cell confluency were analyzed by using a cell scanner. Shown are the percentage of GFP+ cells (**A**), mean fluorescence intensity of GFP+ cells (**B**), quantitative value of GFP expression which is defined as the product of the percentage of GFP+ cells and the mean fluorescence intensity of these cells (**C**), and cell confluency (**D**). The progeny viruses were harvested at 6 days post infection and titrated on LMH-C3532 cells (**E**). ns: not significant, * *p* < 0.05, ** *p* < 0.01, and *** *p* < 0.001.

We compared progeny virus yields from CF1K-CtG-infected LMH-C3532 and LMH-tetR24 cells. As shown in Figure 6A, at 36 h post infection, percentages of GFP+ cells reached 15%, 59% and 75% in LMH-C3532 cells when being infected with MOIs of 4, 40 and 400 vp/cell, while the corresponding values were 0.2%, 6.3% and 57% in LMH-tetR24 cells. Moreover, the mean fluorescence intensity of GFP+ cells was constant in infected LMH-tetR24 cells, and it was always significantly lower than that in infected LMH-C3532 cells, especially at the higher MOIs (Figure 6B). The GFP expression level was compared quantitatively between these two cell lines (Figure 6C), indicating that tetR protein in LMH-tetR24 cells suppressed GFP expressing. Virus replication hindered cell growth, and the cell confluency was significantly lower when being infected at a higher MOI of 400 vp/cell than that at a lower MOI of 40 or 4 vp/cell for both cell lines (Figure 6D). Interestingly, the cell confluency of LMH-tetR24 was approximately two times as high as that of LMH-C3532 when being infected at an MOI of 400 vp/cell, which suggested that very high expression of GFP was detrimental to cell viability.

At 6 days post infection, progeny viruses were harvested and titrated. As shown in Figure 6E, when being infected at MOIs of 0.4, 4 or 40 vp/cell, the titers of progeny viruses produced in LMH-tetR24 reached 3 × 10^9^ IU/mL, which were 4, 10 or 10 times as high as that in LMH-C3532 cells.

### 3.7. Evaluate the Replication of CF1K-CtHA in LMH-tetR24 Cells

**Figure 7:**
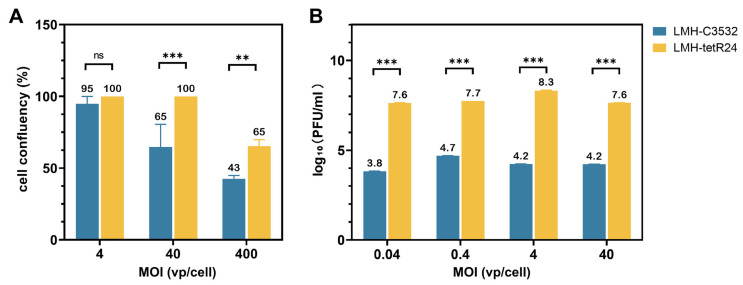
Amplification of FAdV4-CF1K-CtHA in LMH-C3532 and LMH-tetR24 cells. Cells were infected with purified FAdV4-CF1K-CtHA at serial MOIs. At 36 h post infection, cell confluency was analyzed by using a cell scanner (**A**). The progeny viruses were harvested at 6 days post infection and titrated on LMH-C3532 cells (**B**). ns: not significant, ** *p* < 0.01, and *** *p* < 0.001.

Similarly, purified CF1K-CtHA was used to infect LMH-C3532 or LMH-tetR24 cells at serial MOIs, cell confluency was determined at 36 h post infection, and progeny viruses were harvested at 6 dpi and titrated. At 36 h post infection, LMH-tetR24 cells grew to complete confluency when being infected at MOIs of 4 or 40 vp/cell. At the lowest MOI, the confluency of LMH-C3532 was 95%, which is very close to that of LMH-tetR24. However, at the MOIs of 40 vp/cell, the confluency values of LMH-C3532 were 65%, which were significantly lower than that of LMH-tetR24 (Figure 7A). If we compared the data to that from LMH-C3532 infected by CF1K-CtG (Figure 6A), it could be inferred that the infection efficiency was about 59% when LMH-C3532 was infected by CF1K-CtHA viruses at an MOI of 40 vp/cell. If the virus carried a harmless gene, such as GFP, LMH-C3532 cells grew to a confluency as high as 94% (Figure 6D). However, when the virus carried HA gene from influenza virus H5N1, the confluency could hardly reach 65% (Figure 7A), indicating that the expression of HA gene was detrimental and inhibited cell growth. Interestingly, if the expression of HA was suppressed, for example, in LMH-tetR24 cells, the cell still grew to a confluency close to 100%. The cell confluency values went down for both cells when being infected at an MOI as high as 400 vp/cell, suggesting that virus infection inhibited cell growth in this situation. Notably, the confluency of the infected LMH-C3532 cells was significantly lower than that of infected LMH-tetR24 cells at MOIs of 40 or 400 vp/cell, which might result from the combined effects of HA expression and virus replication.

The yields of progeny viruses were dramatically different between infected LMH-C3532 and LMH-tetR24 cells at all tested MOIs, and the ratio of the yield in LMH-tetR24 to that in LMH-C3532 ranged from 1000 to 10000, which resulted from very few progeny viruses produced in LMH-C3532 (Figure 7B).

### 3.8. Inhibition of the Expression of HA in CF1K-CtHA-Infected LMH-tetR24 Cells

**Figure 8:**
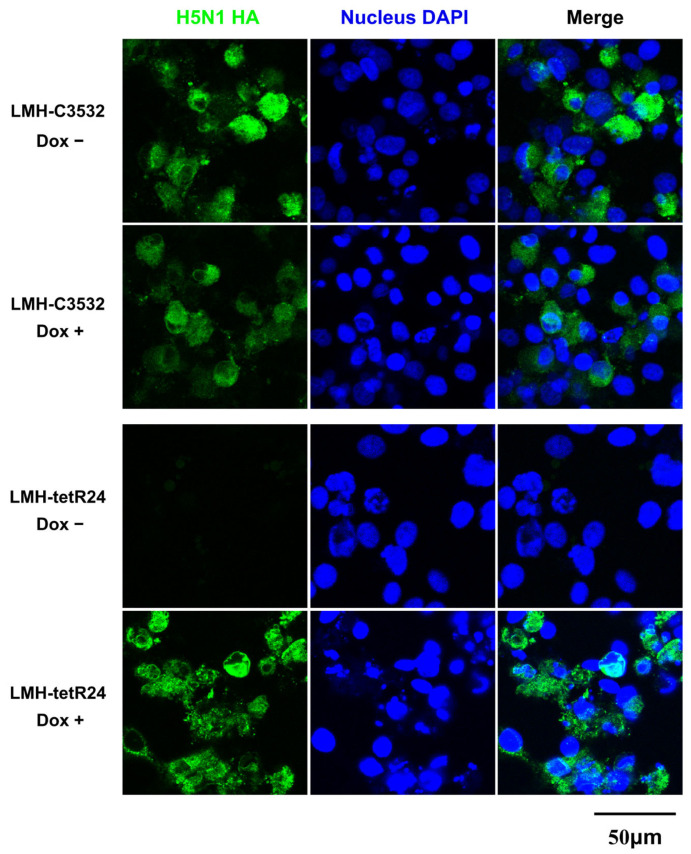
HA expression was inhibited in CF1K-CtHA-infected LMH-tetR24 cells in the absence of Dox. LMH-tetR24 cells were infected with CF1K-CtHA at an MOI of 80 vp/cell for 6 h and cultured in medium with or without 1 µg/mL Dox. At 36 h post infection, the expression of HA was visualized by immunofluorescence assay. LMH-C3532 cells were treated similarly and served as a control.

LMH-C3532 or LMH-tetR24 cells was infected with CF1K-CtHA virus and cultured in medium with or without Dox. Immunofluorescence and Western blot were performed to detect the expression of HA protein at 36 h post infection (Figure 7 and Figure S1). It was seen that HA was expressed in CF1K-CtHA-infected LMH-C3532 cells with or without Dox addition. No HA-positive cells were detected in CF1K-CtHA-infected LMH-tetR24 cells, and the HA expression was induced by the addition of Dox (Figure 8). HA expression was visualized as two bands of approximately 70 and 50 kDa in Western blot (Figure S1), and the results were consistent with that of immunofluorescence. These findings indicated that tetR in LMH-tetR24 cells inhibited transgene expression in the absence of the inducer.

### 3.9. Difference in Plaque Size

**Figure 9:**
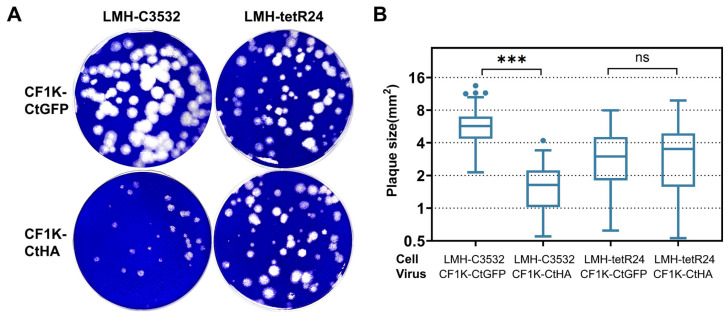
Plaque-forming experiment. LMH-C3532 and LMH-tetR24 cells in 6-well plate were infected with purified CF1K-CtG or CF1K-CtHA viruses and cultured in semi-solid culture medium for 6 days. Plaques were visilized by crystal violet staining after cells were fixed with paraformaldehyde (**A**). Plaque sizes were measured from digital images by using the Fiji image processing package (**B**). ns: not significant, and *** *p* < 0.001.

The sizes of plaques were compared for each cell line after being infected by CF1K-CtG or CF1K-CtHA viruses (Figure 9). CF1K-CtG produced significantly larger plaques on LMH-C3532 cells than CF1K-CtHA did, which suggested that lower yield of progeny viruses in CF1K-CtHA-infected cells resulted in smaller plaques. In contrast, the sizes of plaques generated by these two viruses were comparable when being used to infect LMH-tetR24 cells. This was expected: with transgene expression (GFP or HA) fully suppressed in LMH-tetR24, cell lysis was primarily driven by virus replication and spread. As CF1K-CtG and CF1K-CtHA differed only in their transgenes, the replication and spread characteristics of these FAdV-4 vectors were identical, ultimately yielding plaques of similar size.

### 3.10. Stable tetR Function in LMH-tetR24 Cells After Serial Passaging

**Figure 10:**
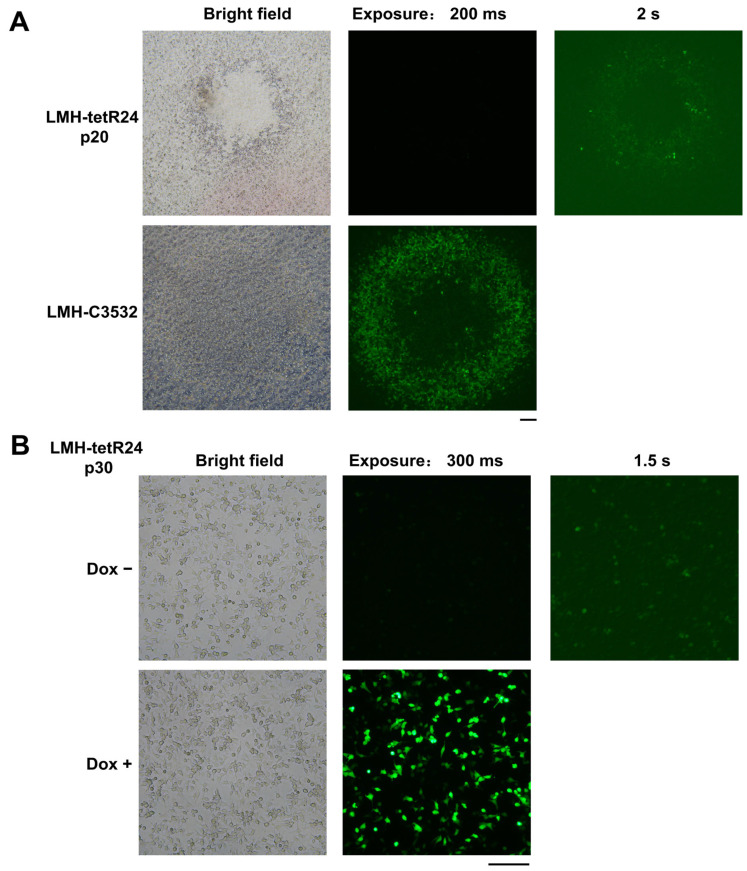
Detection of tetR function in LMH-tetR24 cells after serial passaging. (**A**). LMH-tetR24 passage 20 (p20) was infected with CF1K-CtG and cultivated in semisolid culture medium for 6 days. LMH-C3532 cells were similarly infected and used as a control. (**B**). LMH-tetR24 p30 cells were infected with CF1K-CtG at an MOI of 10 vp/cell and cultivated in culture medium with or without 1 μg/mL Dox for 14 h. Cells were photographed under a fluorescence microscope. Scale bar: 100 μm.

LMH-tetR24 cells were serially passaged, and tetR function was tested by observing GFP fluorescence after CF1K-CtG infection (Figure 10). Plaque-forming experiment was conducted on LMH-tetR24 after 20 passages (p20). It could be seen that CF1K-CtG produced plaques on LMH-tetR24-p20, and the fluorescence of GFP+ cells was so dim that it could only be photographed after long time exposure. In the control group of LMH-C3532 infected with CF1K-CtG, GFP+ cells could be easily observed around the plaques. After 30 passages, LMH-tetR24 cells were infected with CF1K-CtG in the presence or absence of Dox. Addition of Dox induced the expression of GFP. These results demonstrated that LMH-tetR24 retained functional tetR even after 30 passages.

### 3.11. Integration Sites of CDH-EF1a-tetR Provirus in LMH-tetR24 Cells

LMH-tetR24 p20 cells were harvested, and the genomic DNA was extracted and subjected to whole genome sequencing. Six integrating sites of CDH-EF1a-tetR provirus were detected (Table 3).

## 4. Conclusions

In this study, LMH cell line was first adapted for increasing its colony-forming ability, and tetR/tetO regulatory expression elements were utilized to establish a novel FAdV-4 vector system for efficient virus amplification, which consisted of a tetR-expressing packaging cell line and an adenoviral plasmid carrying tetO-bearing CMV promoter for transgene expression.

## 5. Discussion

### 5.1. Supplement of Used Medium (UM) Helps LMH Cells Grow into Monolayer, or Even into Colonies When the Cells Are Seeded at a Low Density

LMH is a cell line extensively used for isolation of avian viruses. As suggested by ATCC, the flask or dishes should be pretreated with gelatin before being used for LMH cultivation. However, we found that LMH cells could not grow into colonies when being seeded at a low density, even on a gelatin-pretreated culture surface. Half-medium change is a standard method to cultivate primary hematopoietic cells, which helps preserve the autocrine growth factors to nourish the cells themselves [18,19]. We were inspired by these strategies. The used medium was collected and added to the medium for LMH cultivation, and the results were expected. If 20–30% UM was added, pretreatment of the flasks with gelatin was unnecessary. This is a helpful finding, considering that gelatin-pretreatment is time-consuming and tedious while addition of UM to the medium is much easier. More importantly, LMH cells could form colonies when being seeded at a low density if UM was supplemented, which made cell adaptation and subclone screening possible.

### 5.2. LMH-C3532 Was an Adapted Subclone of LMH, Which Could Grow into Monolayer or Colonies Without the Addition of UM

Addition of UM is relatively an easier way to cultivate LMH when compared to pretreating flasks with gelatin. However, this approach is still not as convenient as the routine method to cultivate most tumor cell lines. We pursued to exclude the need of UM for LMH culture. LMH-C35 was selected for the characteristics of monolayer growth after two rounds of subcloning of LMH cells in the presence of 30% UM. LMH-C35 cells were then cultivated in fresh medium containing no UM. After experiencing a crisis phase and another two rounds of subcloning, LMH-C3532 cells were obtained. The yield of progeny viruses was similar between LMH-C3532 and the parent cell line of LMH after being infected with a previously constructed FAdV-4 vector, FAdV4-CG. LMH-C3532 is the ideal cell line for our purposes due to its full permissiveness for FAdV-4 vector rescue and amplification, its ease of cultivation, and its colony-forming ability that facilitates genetic modification.

### 5.3. Novel FAdV-4 Vector System Was Established to Package FAdV-4 Vector Carrying Transgene Toxic to Adenovirus Replication

At the beginning, a recombinant FAdV-4 was constructed to carrying CMV promoter-controlled HA gene from a H5N1 influenza virus. The virus was rescued and amplified in LMH-C3532 cells. However, virus purification failed due to low titer of the harvested virus, and we assumed that the low virus yield resulted from high HA expression in packaging cells. Therefore, a new FAdV-4 vector system was substantially needed. In this system, after infecting the packaging cells with a recombinant FAdV-4, the expression of transgene should be minimized so as not to interfere with virus packaging; and when the produced FAdV-4 vector was used to transduce target cells, the transgene would be expressed as expected, without any impairment.

To establish such a novel FAdV-4 adenovirus system, we constructed lentivirus vector CDH-EF1a-tetR to express tetR gene and used it to transduce LMH-C3532 cells, and a subclone, we called LMH-tetR24, was selected after two rounds of functional screening. In the genome of LMH-tetR24, 6 integration sites of provirus of CDH-EF1a-tetR were detected by next generation sequencing (Table 3), ensuring high expression of tetR protein in these cells. Two FAdV-4 vectors (CF1K-CtG or CF1K-CtHA) were constructed to carry transgenes of GFP or HA under the control of tetO-bearing CMV promoter (CMVt). Both viruses could grow to higher titer in LMH-tetR24 than in LMH-C3532 cells. The suppression of transgene expression in LMH-tetR24 cells was very pronounced. The difference in yield of CF1K-CtHA virus between LMH-C3532 and LMH-tetR24 was dramatic, ranging from 3 to 4 orders of magnitude. These results demonstrated that such vector system satisfied the requirement to package adenoviruses carrying transgene that would otherwise severely interfere with virus replication.

The novel FAdV-4 vector system comprised LMH-tetR24 cell line and FAdV-4 adenoviral plasmid pKFAV4-CF1K-CtG. Transgene could be inserted between the dual SwaI sites to replace GFP CDS in pKFAV4-CF1K-CtG to generate a new adenoviral plasmid, in which the expression of transgene will be controlled under tetO-bearing CMV promoter. After linearization with restriction enzyme PmeI, the adenoviral plasmid was used to transfect LMH-tetR24 to rescue recombinant viruses, and LMH-tetR24 cells would also be used for virus amplification. Theoretically, the LMH-tetR24 cells offer an advantage and can be used to package other types of FAdV vector which carry CMVt promoter.

### 5.4. The tetR/tetO System Is the Ideal Strategy for Amplifying Adenoviral Vector Carrying Transgene That Is Toxic to Virus Replication

The Tet-Off and Tet-On systems are widely used to regulate gene expression in eukaryotic cells [20,21]. In these system, the transcription activation domain (AD) of the herpes simplex virus VP16 protein was ligated to TetR, resulting in the tetracycline-controlled transcriptional activator (tTA). Furthermore, a tetracycline-responsive promoter (Ptet) was constructed by fusing 7 tetO sequences to a minimal TATA-box containing eukaryotic promoter, downstream of which the transgene will be located. To make these systems work, both tTA and transgene must be transduced to the same target cell. More sophisticated systems have been developed to achieve lower basal expression of toxic transgenes in the absence of an inducer, as well as higher fold induction upon induction [22]. However, these systems cannot meet the requirement of minimizing transgene expression in adenoviral vector packaging cells while maximizing its expression in target cells.

In 1998, the tetR/tetO system was developed. In this system, original tetR was directly used as the repressor, and 2 tandom tetO sequences (40 bp in length) were positioned 10 bp downstream of the TATAAA element (TATA box) of the CMV major immediate-early promoter (CMVt) for controlling the transcription of transgene. CMVt is different from Ptet in that CMVt works just like CMV promoter in the absence of regulatory protein tetR and will lead to efficient transcription of transgene [12], while Ptet cannot work without tTA. This property makes the tetR/tetO system well-suited for use in adenovirus vector packaging cells. In packaging cells, the expression of transgene is unnecessary and wasteful, because it will consume the cellular resource which otherwise could be used in virus replication. On the other hand, even a transgene that is often considered relatively harmless, such as GFP, may exert cytotoxic effects when overexpressed [23]. Therefore, if adenoviral vector carrying CMVt promoter-controlled transgene is proliferated in tetR-expressing packaging cells, the yield of progeny viruses will be improved. The generated vectors can be directly applied to target cells to express therapeutic gene in gene therapy or antigen protein in vaccination without the need of combination with other vectors. Intriguingly, no antibiotics of tetracycline or doxycycline need to be added throughout the entire process. This strategy has been employed to construct the chimpanzee adenovirus-vectored SARS-CoV-2 vaccine although no clear positive effects were observed [24]. In our study, the yield of FAdV-4 vectors increased when packaging cell of LMH-tetR24 instead of LMH-C3532 was used. In fact, FAdV-4 vector carrying HA gene from a H5N1 influenza virus could only be amplified to a titer high enough for purification when the tetR/tetO system was deployed.

## Figures and Tables

**Table 1:** Summary information of PCR primers.

Fragment	Oligo Name	Sequence	Template	Product (bp)	Aim
EF1a-promoter	2205EF1a-p1	aaaacaaatt acaaaaattc aaaattttat cggtacaagg atctgcgatc gctccggt	pCDH-CMV-MCS-EF1-copGFP	608	construct pCDH-EF1a-tetR
	2205EF1a-p2	ctaaacgaga catggtggcg gctagcgtag gcgccggtca cagcttg			
Lenti-CMVt	2205CDH-CMVtGFP-p1	caaaaacaaa ttacaaaaat tcaaaatttt atcgatacat tgattattg	pcDNA4/TO/Myc-His A	782	construct pCDH-CMVt-GFP
	2205CDH-CMVtGFP-p2	caaaatttta tcgatacatt gattattgac tagttattaa tagtaatca		761	
	2205CDH-CMVtGFP-p3	ggctcgagtt aaacgctaga gtccggaggc tg			
Lenti-GFP	2207CDH-CMVtGFP-p4	cctccggact ctagcgttta actcgagcca ccatggtgag caagg	pLEGFP-C1		construct pCDH-CMVt-GFP
	2207CDH-CMVtGFP-p5	attaccggcg cagatccttg tacattagag tccggacttg tacagct		785	
	2207CDH-CMVtGFP-p6	cacaaatttt gtaatccaga ggttgattac cggcgcagat ccttgtac		810	
FAV-CMVt	2509FV4-CMVtGp1	gtcctttcgt tacagatctt cctacattga ttattgacta gttattaata gtaatca	pCDH-CMVt-GFP	773	construct pKFAV4-CF1K-CtG
	2509FV4-CMVtGp2	catggtggct atttaaatgc tagagtccgg aggctggat			
FAV-GFP	2509FV4-CMVtGp3	gactctagca tttaaatagc caccatggtg agcaagg	pCDH-CMVt-GFP	782	construct pKFAV4-CF1K-CtG
	2509FV4-CMVtGp4	gtggatcgga tatcttatct agatttaaat tagagtccgg acttgtacag ct			
H5N1-tHA	2509FV4-H5N1tHAp1	atccagcctc cggactctag catttaaata gccaccatgg agaacatcgt g	synthesized DNA	1785	construct pKFAV4-CF1K-CtHA
	2509FV4-H5N1tHAp2	ctagatccgg tggatcggat atcttatcta gatt			
WPRE-qPCR	1309WPRE-F1	cctttccggg actttcgctt t	protovirus of lentivirus vectors	176	detect integrated protovirus
	1309WPRE-R1	gcagaatcca ggtggcaaca			
	1309WPRE-p	FAM-actcatcgcc gcctgccttg cc-BHQ1			
hRNaseP-qPCR	1309RNaseP-F1	agatttggac ctgcgagcg	genome of human cells	65	determine copy number of cellular genome
	1309RNaseP-R1	gagcggctgt ctccacaagt			
	1309RNaseP-p	HEX-ttctgacctg aaggctctgc gcg-BHQ2			
cActB-qPCR	1905C-ActBf	tgtggtggtg aagctgtagc ct	genome or cDNA of chicken cells	90	determine copy number of cellular genome
	1905C-ActBr	gccatcctcc gtctggatct			
	1905C-ActBp	HEX-ccgtcaggtc acggccagcc-BHQ2			
tetR-qPCR	2606tetRf	ataagcgtgc tctgctcgat	cDNA of LMH-tetR24	88	determine transcription of tetR
	2606tetRr	tgccaggact ccccttctaa			

**Table 2:** Summary of purified FAdV-4 vectors.

Virus Name	Short Name	Physical Titer (×10^12^ vp/mL)	Infectivity Titer (IU/mL)	Volume (mL)
FAdV4-CF1K-CtG	CF1K-CtG	4.1	2.1 × 10^11^	0.5
FAdV4-CF1K-CtHA	CF1K-CtHA	4.2	6.0 × 10^10^	0.6

note: The raw viruses were harvested from LMH-tetR24 cells cultured in eight 15-cm dishes.

**Table 3:** Integration sites of CDH-EF1a-tetR provirus in the genome of LMH-tetR24 cells.

Chromosome Number	GenBank Accession Number	Insertion Position (bp)	Insertion Direction	Gene ID	Gene Name	Gene Site
chromosome 1	NC_052532.1	180319344-348	reverse	418970	SLC35F2 (solute carrier family 35 member F2)	intron
chromosome 3	NC_052534.1	102060058-062	reverse	396535	APOB (apolipoprotein B)	exon-intron junction
chromosome 4	NC_052535.1	83421715-720	reverse	422900	TMEM129 (transmembrane protein 129)	intron
chromosome 5	NC_052536.1	25584543-548	reverse	423243	TMEM62 (transmembrane protein 62)	intron
chromosome 5	NC_052536.1	33407007-7156	-	-	-	-
chromosome 7	NC_052538.1	17596711-715	forward	424151	DYNC1I2 (dynein cytoplasmic 1 intermediate chain 2)	intron

## Data Availability

The original contributions presented in this study are included in the article/Supplementary Materials. Further inquiries can be directed to the corresponding author.

## Supplementary Materials

The following supporting information can be downloaded at: https://www.mdpi.com/article/10.3390/mps9030100/s1.

## References

[1] Nemerow G., Flint J. (2019). Lessons learned from adenovirus (1970–2019). FEBS Lett..

[2] Greber U.F., Gomez-Gonzalez A. (2021). Adenovirus—A blueprint for gene delivery. Curr. Opin. Virol..

[3] Sakurai F., Tachibana M., Mizuguchi H. (2022). Adenovirus vector-based vaccine for infectious diseases. Drug Metab. Pharmacokinet..

[4] Sampson A.T., Hlaváč M., Gillman A.C.T., Douradinha B., Gilbert S.C. (2025). Developing the next-generation of adenoviral vector vaccines. Hum. Vaccin. Immunother..

[5] Trivedi P.D., Byrne B.J., Corti M. (2023). Evolving Horizons: Adenovirus Vectors’ Timeless Influence on Cancer, Gene Therapy and Vaccines. Viruses.

[6] Park A., Lee J.Y. (2024). Adenoviral Vector System: A Comprehensive Overview of Constructions, Therapeutic Applications and Host Responses. J. Microbiol..

[7] Gao J., Mese K., Bunz O., Ehrhardt A. (2019). State-of-the-art human adenovirus vectorology for therapeutic approaches. FEBS Lett..

[8] Alhashimi M., Elkashif A., Sayedahmed E.E., Mittal S.K. (2021). Nonhuman Adenoviral Vector-Based Platforms and Their Utility in Designing Next Generation of Vaccines for Infectious Diseases. Viruses.

[9] Yan B., Zou X., Liu X., Zhao J., Zhang W., Guo X., Wang M., Lv Y., Lu Z. (2020). User-Friendly Reverse Genetics System for Modification of the Right End of Fowl Adenovirus 4 Genome. Viruses.

[10] Sun Y., Zou X., Guo X., Yang C., Hung T., Lu Z. (2022). CELO Fiber1 Knob Is a Promising Candidate to Modify the Tropism of Adenoviral Vectors. Genes.

[11] Berens C., Hillen W. (2003). Gene regulation by tetracyclines. Constraints of resistance regulation in bacteria shape TetR for application in eukaryotes. Eur. J. Biochem..

[12] Yao F., Svensjö T., Winkler T., Lu M., Eriksson C., Eriksson E. (1998). Tetracycline repressor, tetR, rather than the tetR-mammalian cell transcription factor fusion derivatives, regulates inducible gene expression in mammalian cells. Hum. Gene Ther..

[13] Diez M., Medina-Muñoz S.G., Castellano L.A., da Silva Pescador G., Wu Q., Bazzini A.A. (2022). iCodon customizes gene expression based on the codon composition. Sci. Rep..

[14] Luo W., Yang H., Rathbun K., Pau C.-P., Ou C.-Y. (2005). Detection of human immunodeficiency virus type 1 DNA in dried blood spots by a duplex real-time PCR assay. J. Clin. Microbiol..

[15] Kutner R.H., Zhang X.-Y., Reiser J. (2009). Production, concentration and titration of pseudotyped HIV-1-based lentiviral vectors. Nat. Protoc..

[16] McFarland D.C. (2000). Preparation of pure cell cultures by cloning. Methods Cell Sci..

[17] Ewels P.A., Peltzer A., Fillinger S., Patel H., Alneberg J., Wilm A., Garcia M.U., Di Tommaso P., Nahnsen S. (2020). The nf-core framework for community-curated bioinformatics pipelines. Nat. Biotechnol..

[18] Nafria M., Bonifer C., Stanley E.G., Ng E.S., Elefanty A.G. (2020). Protocol for the Generation of Definitive Hematopoietic Progenitors from Human Pluripotent Stem Cells. STAR Protoc..

[19] Kobayashi H., Takubo K. (2020). Protocol for the Maintenance of Quiescent Murine Hematopoietic Stem Cells. STAR Protoc..

[20] Zhou X., Vink M., Klaver B., Berkhout B., Das A.T. (2006). Optimization of the Tet-On system for regulated gene expression through viral evolution. Gene Ther..

[21] Loew R., Heinz N., Hampf M., Bujard H., Gossen M. (2010). Improved Tet-responsive promoters with minimized background expression. BMC Biotechnol..

[22] De Carluccio G., Fusco V., di Bernardo D. (2024). Engineering a synthetic gene circuit for high-performance inducible expression in mammalian systems. Nat. Commun..

[23] Ganini D., Leinisch F., Kumar A., Jiang J., Tokar E.J., Malone C.C., Petrovich R.M., Mason R.P. (2017). Fluorescent Proteins Such as eGFP Lead to Catalytic Oxidative Stress in Cells. Redox Biol..

[24] Joe C.C.D., Jiang J., Linke T., Li Y., Fedosyuk S., Gupta G., Berg A., Segireddy R.R., Mainwaring D., Joshi A. (2022). Manufacturing a chimpanzee adenovirus-vectored SARS-CoV-2 vaccine to meet global needs. Biotechnol. Bioeng..

