# Supplementary material for: An Efficient TetR/TetO-Integrated Packaging System for Fowl Adenovirus 4 Vector Carrying Toxic Transgene
Source: Methods Protoc. 2026 Jun 22;9(3):100. doi: 10.3390/mps9030100 (PMC13306192; doi:10.3390/mps9030100)
Supplement: Supplementary file 1 [file mps-09-00100-s001.zip › mps-4327099-supplementary.pdf]

methods and protocols

MDPI

Supplementary Materials

synthesized tetR DNA sequence (657 bp)

```
  1 atgtctcgtt tagataagtc taaggtgatt aattccgcat tagagctgct taatgaagtc

 61 ggaatcgaag gcctgaccac ccgtaaactc gcccagaagc tgggtgtcga gcagcctacc

121 ttgtattggc atgtaaaaaa taagcgtgct ctgctcgatg ctttagctat tgagatgctg

181 gatagacacc atacccactt ttgtccttta gaaggggagt cctggcaaga ttttctgcgt

241 aataacgcta agagttttcg ttgtgcttta ctgagtcacc gcgatggagc taaagtccat

301 cttggaacac gtcctacaga aaagcagtat gaaaccctgg aaaatcagct cgcctttctg

361 tgtcagcagg gtttttctct tgagaatgca ttatacgctc tttccgctgt cggtcatttt

421 accttaggat gcgtcttgga agatcaagag catcaagtcg ctaaagagga acgtgaaaca

481 cctaccaccg attccatgcc gcctctgctt cgtcaggcta tcgagttatt tgatcaccag

541 ggagcagaac cagccttcct tttcggcctt gaactgatca tctgtggatt agaaaaacaa

601 ctgaaatgtg aatctgggtc cgcatactcc ggatcccgtg aattccgttc ttactaa
//
```

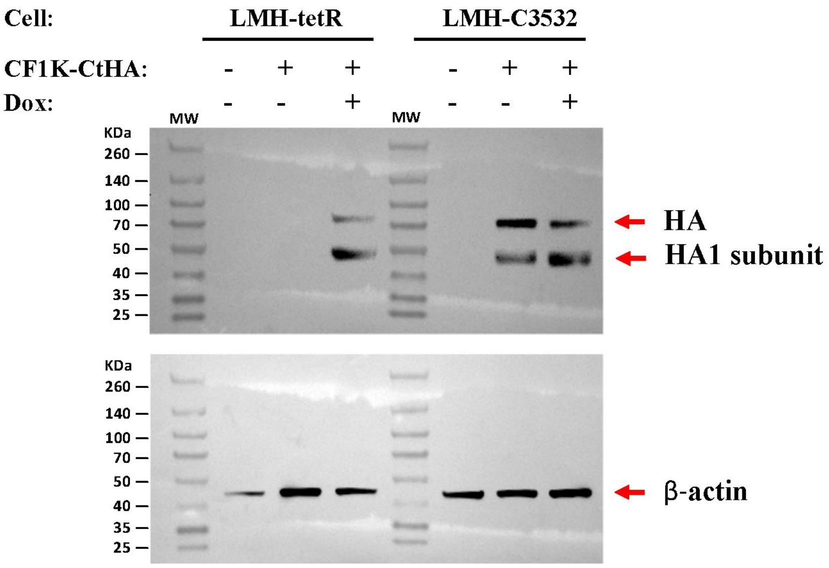

Figure S1. Detection of HA expression in CF1K-CtHA-infected LMH-tetR24 cells by Western blot. LMH-tetR24 cells were infected with CF1K-CtHA for 6 hours at an MOI of 40 vp/cell and cultured in medium with or without 1 μg/ml Dox. At 36 hours post infection, the total cell proteins were extracted and the expression of HA was detected by Western blot with 1:5000 diluted anti-HA (Influenza A H5N1) primary antibody (Cat. 11048-RM09, SinoBiological, Beijing, China). The membrane was stripped and reprobed with anti-β-actin antibody (Mouse Anti-β-actin mAb, Cat. TA-09, ZSGB-Bio, Beijing, China) to show the protein loading across all lanes. LMH-C3532 cells were treated similarly and served as a control. The molecular weights calculated from amino acid sequences are 64.1, 37.3 and 41.7 kDa for HA, HA1, and β-actin, respectively. MW: molecular weight marker (Spectra Multicolor Broad Range Protein Ladder, Cat. 26634, Thermo Fisher Scientific, Waltham, MA, USA).

https://doi.org/10.3390/xxxxx
